# Sleep need outcompetes preparation: reframing sleep initiation through naturalistic behavior

**DOI:** 10.1093/sleep/zsag178

**Published:** 2026-06-28

**Authors:** Rhîannan H Williams, Bastiaan Van der Veen

**Affiliations:** Boehringer Ingelheim Pharma GmbH & Co. KG, Neuroscience + Mental Health, Biberach an der Riß, Germany; Boehringer Ingelheim Pharma GmbH & Co. KG, Neuroscience + Mental Health, Biberach an der Riß, Germany

## Abstract

**Graphical Abstract:**
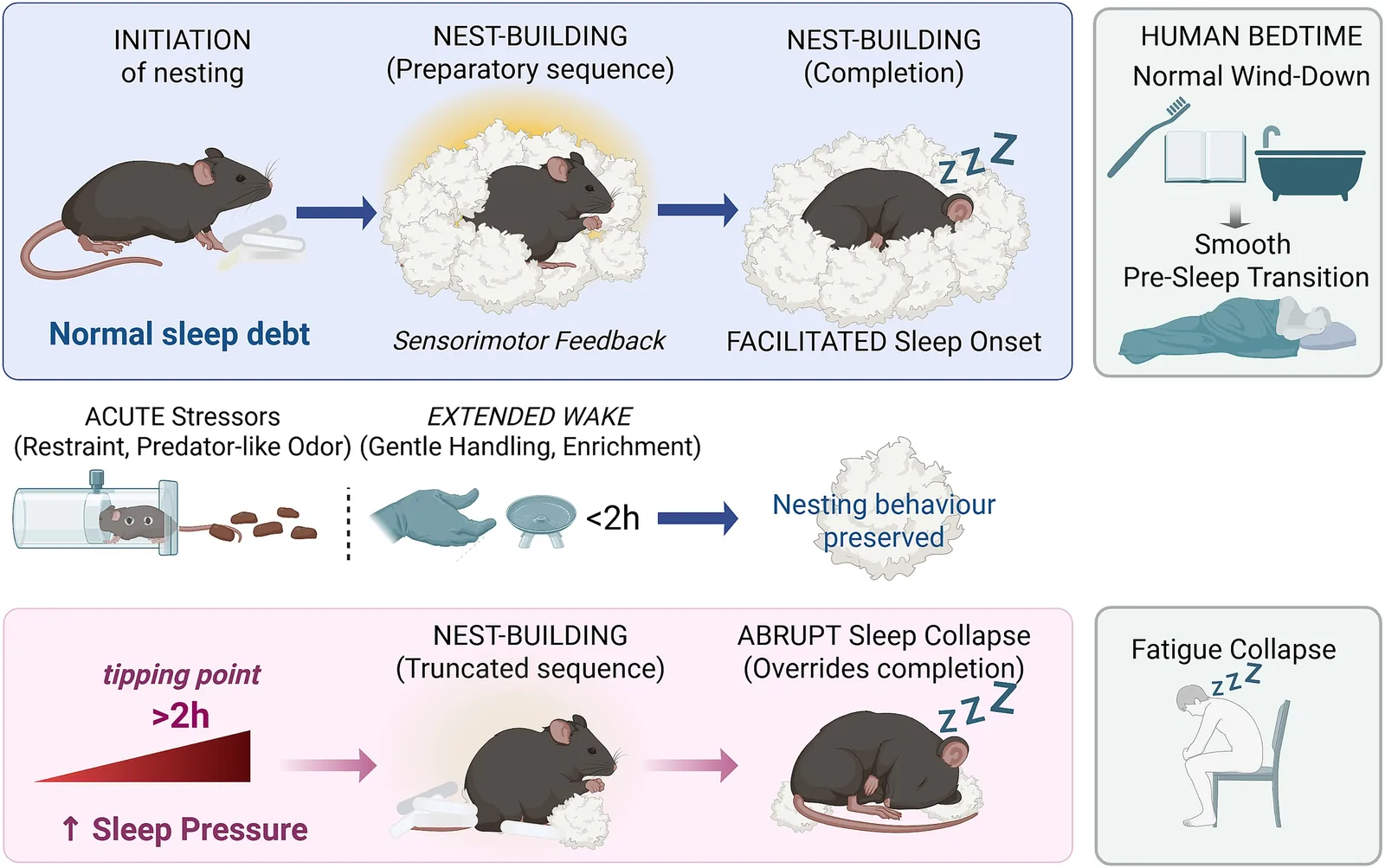

Sleep research has defined wake, Non-Rapid Eye Movement (NREM), and Rapid Eye Movement (REM) states and many of their governing circuits with great precision. Yet the behavioral processes that lead into sleep—and integrate sensory and environmental context—remain less well understood. Building on earlier work [1], Freitag et al. [2] address this gap by treating pre-sleep behavior as an actively regulated and biologically meaningful component of sleep initiation. Their key conceptual advance is that sleep onset is not simply a switch between brain states, but a coordinated behavioral transition that reflects circuit function at the level of the whole organism.

The authors show that pre-sleep behavior—operationalized here as nest-building in mice—forms a distinct and regulated behavioral domain rather than a simple byproduct of rising sleepiness. The central finding is that once sleep deprivation exceeds a critical threshold (>2 h), mice truncate their normal nest-building routine and fall asleep rapidly. This pattern is similar after 2, 4, or 6 h of prior wakefulness, indicating that greater sleep debt increases homeostatic pressure for sleep without proportionally reducing nest-building further. Nest-building is often initiated but not sustained, suggesting that completion of this behavior is governed by mechanisms not fully explained by classical sleep homeostasis. Rather than a graded decline, the data point to a rapid re-prioritization: as sleep pressure rises, behavioral resources shift away from goal-directed preparation toward immediate physiological recovery.

Freitag et al. [2] also reveal a circadian dimension to sleep preparation. Mice engaged more strongly in nest-building during the light phase, when sleep propensity is high, and showed little interest at the start of the active dark phase, when sleep need is low. Pre-sleep nest-building therefore appears to be gated by circadian timing, not driven solely by accumulated sleep pressure. Mice also spent substantial time in the nest before sleep onset (~30 min), raising the possibility that nest occupancy itself may contribute sensory or contextual signals that facilitate sleep initiation. It will be important to test how this behavioral gating changes under circadian disruption, such as shifted light schedules or simulated jet lag.

Crucially, the suppression of nesting was not explained by general stress or by the specific deprivation method. Mice exposed to acute stressors—30 min restraint or predator-associated olfactory cues—still built higher-quality nests, arguing against anxiety or gross motor disruption as the primary cause of the sleep-loss effect. Likewise, both gentle handling and enriched-environment deprivation produced the same marked reduction in nest-building. These controls support the conclusion that sleep loss itself truncates this behavioral domain. That said, deprivation methods are not biologically neutral: handling- and novelty-based paradigms can differ in stress burden and in the rebound sleep they produce [3, 4]. Individual variability in nesting may therefore still reflect differences in stress sensitivity, and future work could strengthen this point by relating nesting scores to corticosterone measures.

Methodologically, the study is strong because it combines automated video tracking, manual behavioral annotation, and electroencephalogram/electromyography (EEG/EMG) measures to capture sleep initiation at both behavioral and physiological levels. An important advance is the repositioning of nest-building from a welfare readout [5] to a functional assay of sleep readiness. This has clear translational relevance. In humans, pre-sleep routines help facilitate sleep onset, and their disruption is associated with insomnia and poor sleep quality [6–10]. Naturalistic behavioral measures may therefore reveal vulnerabilities in sleep regulation that standard metrics such as sleep latency or EEG spectral power miss. The apparent behavioral “tipping point” between 1 and 2 h of extended wakefulness in mice also raises the possibility that analogous thresholds in humans could prove informative for fatigue-related risk.

Despite its strengths, several limitations remain. First, nest quality is only a coarse proxy for “motivation,” because it combines initiation, persistence, and completion into a single outcome. The pattern observed here is also not easily explained by a simple graded motivational model. Instead, nest-building appears to collapse once sleep pressure passes a modest threshold and then remains suppressed despite further increases in sleep need. A more plausible interpretation is that rising sleep pressure limits the capacity to sustain goal-directed behavior, forcing rapid re-prioritization toward sleep. In this view, the critical issue is not only the drive to act, but whether the animal can remain awake long enough to execute the preparatory sequence. Second, the experiments were performed in singly housed mice under simplified conditions; social context, hunger, or threat could alter the balance between nest preparation and sleep in more natural settings. Third, an informative control is missing: no nest manipulation after 6 h of sleep deprivation would have helped place the findings in context. A low-effort nesting condition could also test whether sleep deprivation reduces motivation or simply the capacity to complete the behavior. Finally, although the authors discuss candidate circuits, the study remains largely phenomenological. Direct recording or manipulation of specific neurons during the pre-sleep phase—especially under sleep deprivation—will be needed to define how sleep, arousal, and behavioral preparation interact.

Ultimately, the study’s main contribution is conceptual. By placing pre-sleep behavior at centre stage, Freitag et al. [2] encourage us to view sleep initiation as an integrative, whole-organism process rather than a purely neural state change. This perspective is especially important for translational research, where human sleep habits and preparatory routines are only partly captured in animal models. Their findings show that sleep loss can collapse the normal behavioral lead-up to sleep, underscoring that we must consider not only how sleep is generated, but also how it is approached. For researchers working across sleep homeostasis, circadian biology, and translation, these pre-sleep behaviors may prove to be sensitive readouts of circuit integrity and useful entry points for understanding sleep disorders.
